# Performance of TB-LAMP in the Diagnosis of Tuberculous Empyema Using Samples Obtained From Pleural Decortication

**DOI:** 10.3389/fmed.2022.879772

**Published:** 2022-06-29

**Authors:** Chang Liu, Lichao Fan, Jiansong Zhang, Qi Hong, Yi Ren, Huaiyu Tian, Yu Chen

**Affiliations:** ^1^Department of Thoracic Surgery, Shenyang Tenth People's Hospital, Shenyang Chest Hospital, Shenyang, China; ^2^Department of Tuberculosis, Shenyang Tenth People's Hospital, Shenyang Chest Hospital, Shenyang, China

**Keywords:** Mycobacterium tuberculosis, TB empyema, pleural tissue specimens, loop-mediated isothermal amplification assay (LAMP), diagnostic study

## Abstract

**Purpose:**

To evaluate the performance of TB-LAMP in the diagnosis of TB empyema using pleural tissue specimens obtained during pleural decortication.

**Methods:**

Using the clinical records and the different diagnostic test results of patients who underwent pleural decortication in a TB-designated hospital over 3.5 years, we calculated the sensitivity, specificity positive predictive, and negative predictive values of the pathology, MGIT 960 culture, and TB-LAMP obtained by using pleural tissue specimens against the etiologic diagnosis and composite clinical reference standard (CCRS) as the reference standards.

**Result:**

A total of 304 patients' records were extracted. All these patients had gone through pleural decortication. When the etiologic diagnosis was used as the reference, the sensitivity of TB-LAMP in identifying TB empyema was 77.8% (compared to 10.6% of MGIT 960 *P* < 0.05). The sensitivity of MGIT 960, pathology, and TB-LAMP was 8.2%, 77.7%, and 67.2% against CCRS as the reference; and the specificity of the three was 100.0, 100.0, and 96.2% against the same standard. A combination of pathology and TB-LAMP would increase the sensitivity and specificity to 84.7 and 96.0%. Using TB-LAMP to diagnose TB empyema using pleural tissue samples obtained from pleural decortication was faster with satisfactory performance.

**Conclusion:**

TB-LAMP has great potential in faster and more accurate diagnosis of TB empyema. Our findings provide insights for optimizing diagnostic algorithms for TB empyema.

## Introduction

Tuberculosis (TB) empyema remains a dangerous situation with significant mortality and morbidity (1, 2). There are three stages of TB empyema: stage I is an exudative phase; the second stage is the fibrinopurulent phase, also called stage II empyema, and the third stage is the consolidation phase (3). The definitive diagnosis of TB empyema depends on imaging evidence as well as histological or microbiological identification of the Mycobacterium tuberculosis (MTB) in the targeted samples (4). However, microbiological confirmation is often hard given the low bacterial loads and the difficulty in obtaining appropriate pleural effusion or tissue samples. A few previous research has estimated that in the process of video-assisted thoracoscopic surgery (VATS), only 25% of the culture obtained from patients confirmed of stage III TB empyema was positive (3). Because of the limited value of microscopy and culture methods for TB detection in TB empyema, microbiological confirmation of TB empyema is rare, and clinical diagnosis depends mainly on contact history, clinical symptoms, and chest radiography, or the composite clinical reference standard (CCRS). There are several advantages of surgical management, which could help the doctors to obtain samples for disease diagnosis, help the patients' lungs to re-expand thus improving the recovery; therefore, surgical management is more adopted as a mandatory treatment option for TB empyema, especially for stage II or stage III patients (5). Besides, a large amount of pleura tissue could be obtained from the site of pathology under the vision to increases the diagnostic yield of MTB (6, 7). An early and accurate diagnosis is critical for identifying the appropriate management and better recovery of the patients (8).

There is increasing evidence supporting using molecular assays based on nucleic acid amplification techniques (NAATs) for rapid TB diagnosis (9). The loop-mediated isothermal amplification (LAMP)-based assay, TB-LAMP (Tokyo, Japan), as one of the NAATs, became a rapid diagnostic test for pulmonary TB, as recommended by the World Health Organization (10). We now report the performance of TB-LAMP in the diagnosis of TB empyema using pleural tissue samples obtained from pleural decortication against MGIT 960 as a reference. In addition, we also evaluated our results against CCRS as a reference to provide a more comprehensive evaluation of the different methods.

## Methods

### Study Setting and Population

This retrospective study was performed in Shenyang Tenth People's Hospital, also called Shenyang Chest Hospital using patients' records from January 2017 to June 2021. All the records of patients who had both pleural decortication and TB-LAMP results were enrolled. The stage II/III empyema criterion included symptoms such as fever, chest pain, hemoptysis; a chest CT scan consistent with empyema; if pleural fluid could be collected before surgery, effusion biochemical characteristics record with pH <7.2 and LDH>1,000 U/L and Glc <40 mg/dl. Surgical treatment was necessary for patients with pleural peel encasing the lung (trapped lung), multiloculated empyema, inadequate drainage of empyema despite chest tube, and persistent bronchopleural fistula with a collapsed lung. Given that patients who had anti-tuberculosis treatment may be wrongly diagnosed as active TB patients (11), we excluded patients who were taking anti-TB treatment. Patients were also excluded if they were HIV positive.

Two distinctive standards were used to evaluate the performance of the different tests. The etiological diagnosis, which was the conventional gold standard, included the isolation of MTB from pleural tissue samples by culture or pathology demonstrating caseating granuloma. The etiological diagnosis was used as the first reference standard to check the performance of TB-LAMP. Given that culture and pathology are suboptimal in detecting TB empyema, CCRS was also used as a standard for comparison, as recommended in the literature in the absence of an ideal gold standard (12, 13), which included the following items: (1) etiologically confirmed tuberculosis (as listed in the first reference standard), (2) positive for MTB culture or Xpert of MTB using pleural fluid samples, (3) clinical symptoms or signs indicating TB plus any one of the following indicators: a positive TB skin test or interferon-gamma release assay (IGRA), improvement in chest images, or significant improvement in the clinical symptoms after anti-TB chemotherapy.

### Pleural Decortication Procedure

All the patients included in this study had pleural decortication (either through VATS or open surgery) in this TB-designated hospital. The incision into the chest cavity was through the sixth intercostal space anterior to the posterior axillary line. The lung is able to re-expand by cleaning the cavity pus and the pleural effusion. Abnormal lesion biopsy pieces were taken from the parietal and visceral pleura and diaphragm surface. Up to 10 biopsy pieces were obtained. Tissue samples from multiple pleural lesions were also collected for Mycobacterium culture and TB-LAMP assay. The rest of the specimens were sent to the pathology department for pathological examination.

### Sample Preparation and Diagnostic Tests

#### Specimen Preparation

The mixed pleural sample was first minced for the detection of MTB and transferred to a 2-mL sample tube with sterile ceramic beads adding a diameter of 6 mm, and 0.5 to 1.0 mL of 0.9% sodium chloride for injection. After tightening the lid, the sample tube was embedded into a FastPrep®-24 homogenizer (MP Biomedicals, USA), with a frequency of 6.5 m/s, time to 20 s, and general tissue oscillation to two to three times. The prepared suspension was then used for MGIT 960 culture and TB-LAMP tests.

#### MGIT 960 Culture

After specimen preparation, 0.5 mL suspension was taken and placed in MGIT 960 liquid culture tube and incubated at 37°C for 42 days. If the instrument reported a positive result on or before day 42, the culture would be smeared for microscopy, and 0.1 mL of the Auramine O dye was added for Ziehl-Neelsen staining, followed by fluorescence microscopy. True positive would be considered if acid-fast bacilli (AFB) were detected. However, for those samples with no AFB, the results would be considered negative. All the test steps followed the MGIT 960 manual (14).

#### TB-LAMP Assay

TB-LAMP assay was carried out according to the WHO guideline (10). Loopamp Procedure for Ultra Rapid Extraction (PURE) DNA extraction kit (Eiken Chemical) and Loopamp MTBC Detection Kit (Eiken Chemical) were used during the procedure following the manufacturer's instructions. The prepared 60-μL suspension was placed in a heating tube and incubated at 90°C for 5 min. After that, the sample was mixed well with absorption powder. A 30-μL DNA solution was extracted and placed in a reaction tube, and loop-mediated isothermal amplification was performed at 67°C for 40 min. After the reaction, the tube was put into the fluorescence visual detection unit to observe the reaction results. The whole detection process can be completed within 1 h.

### Statistical Analysis

The values and their 95% confidence intervals of the following parameters were calculated using the Wilson binomial method with etiological diagnosis as the reference standard: sensitivity, specificity, positive predictive values (PPV), and negative predictive values (NPV) of Mycobacterial culture and TB-LAMP. After that, the above-mentioned parameters were calculated for a second time, using CCRS as the reference standard together pathology and the different combinations of them. Differences between any two assays or their combinations were assessed using a one-way analysis of variance (ANOVA). If the *P-*value (two-tailed) is <0.05 the result would be considered statistically significant. All statistical analyses were carried out using SPSS 20.0 and Excel 2010.

## Results

### Demographical Information of the Participants

**Figure 1:**
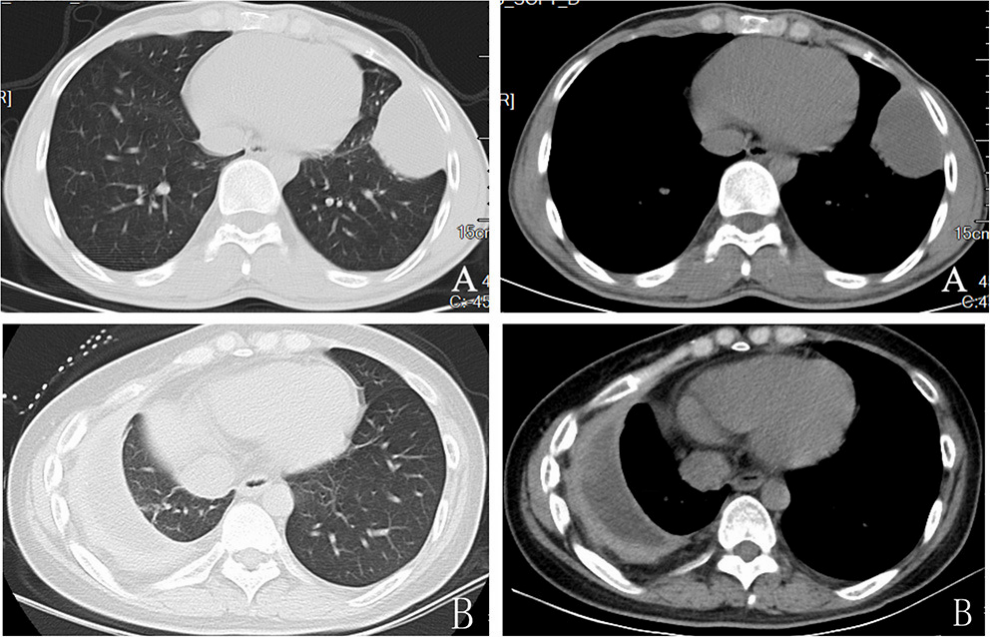
Preoperative computed tomographic scan showing thickened parietal and visceral pleura with collection.Stage II ertuberculosis empyema **(A)**and stage III tuberculosis empyema **(B)**.

**Figure 2:**
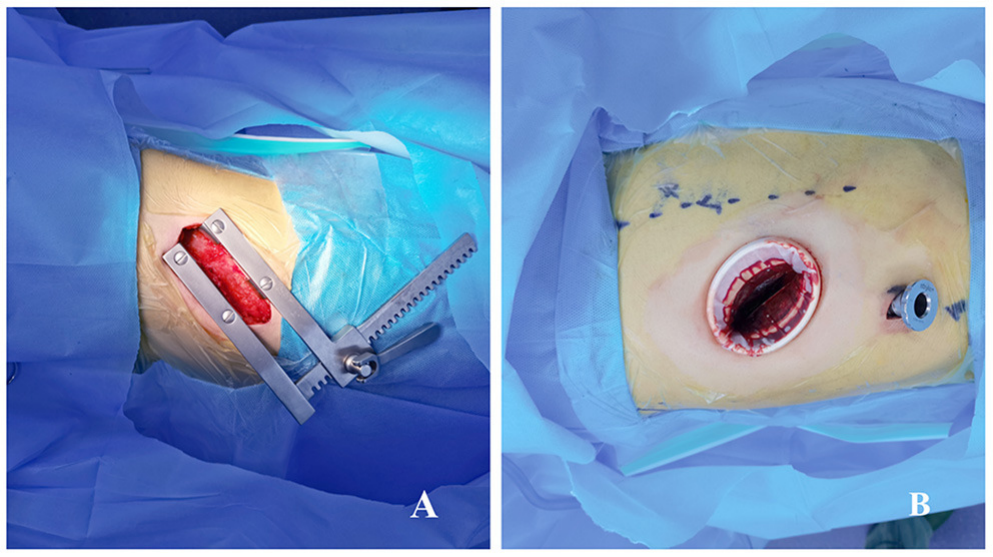
Image showing port positions for open surgery decortication **(A)** and video-assisted thoracoscopic surgery decortication **(B)**.

**Figure 3:**
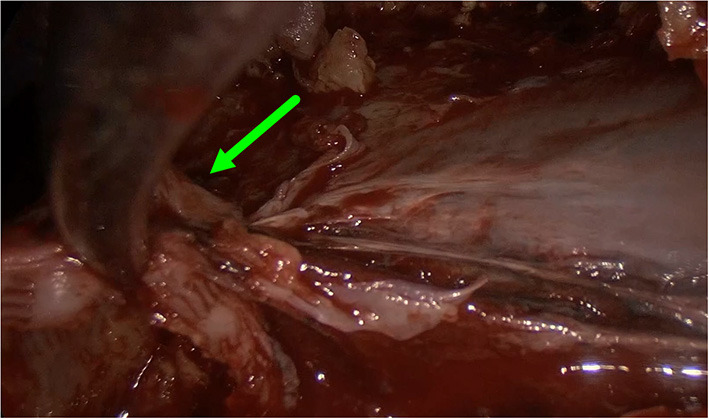
Intraoperative image showing removal of visceral pleura.

**Figure 4:**
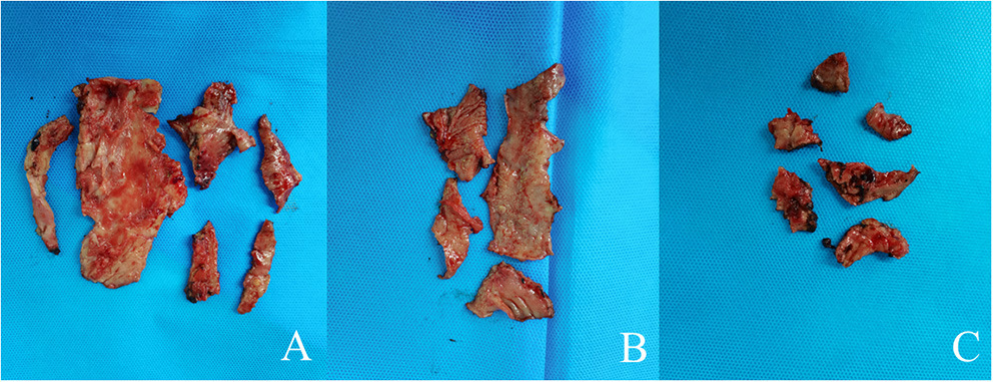
Image showing theparietal pleura **(A)**, the visceral pleura**(B)**, and the diaphragm surface **(C)**.

We enrolled a total of 304 patients suspected of TB empyema and had pleural decortication in this study. The participants' demographical information could be found in Table 1. among which 220 patients (72.4%) were male and 84 patients (27.6%) were female. Two hundred thirty-four patients (76.9%) were suspected of contracting TB empyema for the first time, indicating no previous anti-TB therapy. CT imaging showed different stages of TB empyema, such as diffuse thickening of both the visceral and parietal pleura, subpleural abscess, separated by fluid (the split-pleura sign) (Figure 1). Stage II (a transitional or fibrinopurulent phase) 29 (9.5%), stage III (an organizing or consolidation phase), 275 (90.5%). Among the patients who underwent pleural decortication, 107 (35.2%) patients underwent VATS and 197 (64.8%) patients underwent open surgery (Figures 2–4). Two hundred twenty-nine patients were TB empyema according to the CCRS (accounting for 75.3%), and 75 patients with nontuberculous empyema (24.7%): 33 patients with lung cancer (10.8%), 20 patients with bacterial empyema (6.6%), 13 patients with pleural mesothelioma (4.3%), and another nine patients with other conditions (3.0%). Over 85% of the patients were under 65 years old. The average age of the participants was 45.51 ± 16.74 years old, ranging from 6 to 75 years.

**Table 1:** Clinical characteristics of the patients (*N* = 304).

**Variable**	**No. (%)**
**Gender**	
Male	220 (72.4%)
Female	84 (27.6%)
**Age (years old)**	
<65	260 (85.5%)
≥65	44 (14.5%)
**History of anti-TB treatment**	
Yes	70 (23.1%)
No	234 (76.9%)
**Surgical approach**	
VATS	107(35.2%)
Open	197(64.8%)
**Stage of pleural empyema**	
Fibropurulent phase	29 (9.5%)
Organized phase	275 (90.5%)
**Clinical diagnosis**	
TB empyema	229 (75.3%)
Bacterial empyema	20 (6.6%)
Pleural mesothelioma	13 (4.3%)
Lung cancer	33 (10.8%)
Others	9 (3.0%)

*TB, Tuberculosis; VATS, video-assisted thoracic surgery*.

### Performance of TB-LAMP Using Etiology as the Reference

The performance of TB-LAMP for detecting MTB infection in the 304 suspected TB empyema patients (180 positives and 124 negatives) when etiology was used as a reference was presented in (Table 2). TB-LAMP had higher sensitivity than MGIT 960 (77.8 vs.10.6%, *P* < 0.05) but lower specificity than MGIT 960 (86.3 vs. 100.0%, *P* < 0.05).

**Table 2:** Performance of TB-LAMP in the diagnosis of TB empyema with etiology as the reference standard.

**Diagnostic assay**	**Performance parameters [Mean percentage (n/n), 95% CI]**			
	**Sensitivity**	**Specificity**	**PPV**	**NPV**
**Single tests**				
TB-LAMP	77.8% (140/180 70.9%−83.5%)	86.3% (107/124 78.7%−91.6%)	89.2% (140/157 83.0%−93.4%)	72.8% (107/147 64.7%−79.6%)
MGIT 960	10.6% (19/180 6.6%−16.2%)	100% (124/124 96.3%−100%)	100% (19/19 79.1%−100%)	43.5% (124/285 37.7%−49.5%)

*CI, confidence interval; PPV, positive predictive value; NPV, negative predictive value*.

### Performance of TB-LAMP and the Conventional Diagnostic Methods Using CCRS as the Reference

**Figure 5:**
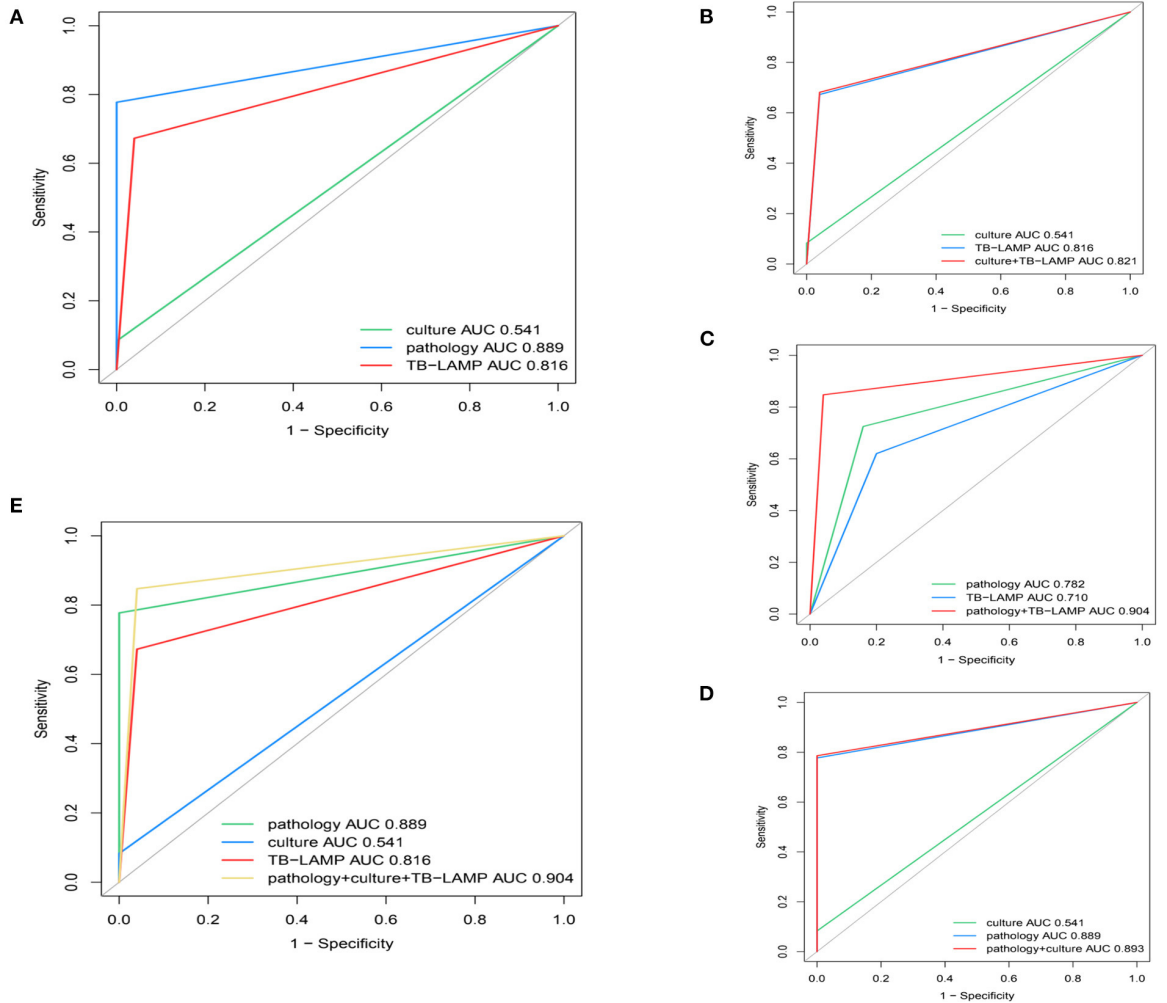
Receiver operating characteristic (ROC) curves for determining the sensitivity and specificity of **(A)** culture, TB-LAMP and pathology using CCRS as a reference **(B)** culture, TB-LAMP and the two tests in parallel using CCRS as a reference **(C)** pathology, TB-LAMP and the two tests in parallel using CCRS as a reference **(D)** pathology, culture and the two tests in parallel using CCRS as a reference **(E)** culture, TB-LAMP, pathology and the three tests in parallel using CCRS as a reference. TB-LAMP: Loop-mediated Isothermal Amplification Assay; CCRS: composite clinical reference standard.

Based on the CCRS criteria, 229 of the 304 patients enrolled in the study had a positive TB empyema diagnosis and 75 negative TB empyema diagnoses, with 19, 178, and 154 cases of TB empyema being identified by mycobacterial culture, pathology, and TB-LAMP, respectively. Results from our assessment of the performance of TB-LAMP, MGIT 960, and pathology alone or in combination for diagnosing TB empyema using clinical diagnosis as a reference are shown in Table 3. The sensitivity of TB-LAMP (67.2%, 95% CI 60.7–73.2%) was tested superior than MGIT 960 (*P* < 0.005) and inferior than pathology (*P* < 0.005) while using CCRS as the reference standard. The specificity of TB-LAMP was 95.6% (72/75), (95% CI: 88.0–99.0%), showing no significant difference than pathology. The intergroup comparison showed no significant difference when stratifiying patients according to anti-TB therapy history and gender.When pathology and TB-LAMP were performed together, the overall sensitivity and specificity were 84.7% (194/229) and 96.0% (72/75) respectively. The combination of TB-LAMP and pathology improved the sensitivities by 7.0% and 17.5% compared to that of pathology or TB-LAMP alone. The AUC of the ROC curve for pathology, TB-LAMP, and the combination of the two was 0.782, 0.710, and 0.904 respectively (Figure 5). When MGIT 960 culture and TB-LAMP were performed together, the sensitivity and specificity were 68.1% (156/229) and 96.0% (72/75) respectively. The combination of TB-LAMP and culture improved sensitivities by 59.9 and 0.4% compared to culture or TB-LAMP alone. The AUC of the ROC curve for culture, TB-LAMP, and the combination of the two was 0.541, 0.816 and 0.821 respectively (Figure 5). The combination of pathology, MGIT 960 and TB-LAMP increased the sensitivity and specificity to 84.7% (194/229) and 96.0% (72/75) respectively, which is in line with the results of using pathology and TB-LAMP together.

**Table 3:** Performance of TB-LAMP, MGIT960 culture and pathology as single tests and combined tests in the diagnosis of TB empyema with CCRS as the reference standard.

**Diagnostic assay**	**Performance parameters [Mean percentage (n/N), 95% CI]**			
	**Sensitivity**	**Specificity**	**PPV**	**NPV**
**Single tests**				
Pathology	77.7% (178/229 71.7%−82.8%)	100% (75/75 93.9%−100%)	100% (178/178 97.4%−100%)	59.5% (75/126 50.4%−68.1%)
MGIT 960	8.2% (19/229 5.2%−12.8%)	100% (75/75 93.3%−100%)	100% (19/19 79.1%−100%)	26.3% (75/285 21.3%−31.9)
TB-LAMP	67.2% (154/229 60.7%−73.2%)	96.0% (72/75 88.0%−99.0%)	98.1% (154/157 94.1%−99.5%)	49.0% (72/147 40.7%−57.3%)
Pathology + MGIT 960	78.6% (189/229 72.6%−83.6%)	100% (75/75 93.9%−100%)	100% (180/180 97.4%−100%)	60.4% (75/124 51.3%−69.0%)
Pathology + TB-LAMP	84.7 (194/229 79.2%−89.0%)	96% (72/75 88.0%−99.0%)	98.5% (194/197 95.3%−99.6%)	67.3% (72/107 57.5%−75.9%)
MGIT 960 + TB-LAMP	68.1% (156/229 61.6%−74.0%)	96% (72/75 88.0%−99.0%)	98.1% (156/159 94.1%−99.5%)	49.7% (72/145 41.3%−58.0%)
Pathology + MGIT 960 + TB-LAMP	84.7 (194/229 79.2%−89.0%)	96% (72/75 88.0%−99.0%)	98.5% (194/197 95.3%−99.6%)	67.3% (72/107 57.5%−75.9%)

*Pleural tissue was used for testing. CI, Confidence interval; PPV, Positive predictive value; NPV, Negative predictive value; CCRS, composite clinical reference standard*.

## Discussion

In this study, we conducted a retrospective study on the performance of TB-LAMP in the diagnosis of TB empyema using pleural tissue specimens obtained during pleural decortication. In the meantime, we also evaluate the performance of MGIT 960 culture and pathology, as well as the different combinations. Our findings indicated that TB-LAMP demonstrated high sensitivity in TB empyema diagnosis using pleural tissue samples. Using pleural tissue samples, the performance of TB-LAMP was significantly better than MGIT 960 culture. TB-LAMP had great potential in the early diagnosis of TB empyema, which was proven to be efficient, precise, and simple. The combination of MGIT 960, pathology, and TB-LAMP demonstrated a possible way to optimize the diagnostic algorithm for stage II/III TB empyema.

Though invasive, the diagnostic value of pleural tissue samples is irreplaceable in patients with tuberculous pleural effusion and stage II/III TB empyema (5). Pleural decortication is a routine treatment for stage II/III TB empyema with or without bronchopleural leakage (15). In patients with tuberculous pleurisy, the detection of MTB in pleural tissue samples was significantly higher than that in pleural effusion samples. Christopher et al. showed the yields of the microbiological tests against histopathology on thoracoscopic biopsy sample and pleural fluid were as followed: Xpert 45% using pleural tissue specimens, Xpert 14% using pleural effusion, mycobacterial culture 39% using pleural tissue specimens, and mycobacterial culture 17% using pleural effusion (6). It could be concluded that pleural tissue specimens yield improved the yield than pleural effusion in both Xpert and mycobacterial culture. Pleural tissue specimen was used in a lot of research through conventional ultrasound-guided or closed pleural biopsies for diagnosis purposes (16). Fan et al. confirmed that the etiological detection rate of pleural tuberculosis on contrast-enhanced ultrasound (CEUS)-guided pleural biopsy specimens were improved (17). The most effective method for obtaining pleural tissue was generally obtained through thoracoscopy under direct vision of the physicians. It has also been shown that thoracoscopic pleural samples improved the yield of Xpert in pleural tuberculosis (15). TB empyema is developed from chronic active infection of the pleura, an infectious process that increased in neutrophils and developed into purulent effusion and eventually extensive pleural thickening and calcification (2). Surgical treatment has become a necessary treatment option for stage II/III TB empyema patients (5). Currently, thoracotomy and VATS are widely used for these disease conditions (18). As far as we know, there is a lack of NAATs based studies using pleural tissue specimens for the diagnosis of stage II/III TB empyema and we hope this study would cover this research gap.

In this retrospective study, the medical records including the test results of 304 patients were used. The sensitivity of TB-LAMP for detecting TB empyema in etiological and CCRS diagnosis standards was 77.8% (140/180) and 67.2% (154/229) respectively. The specificity of TB-LAMP was 86.3% (107/124) and 96% (72/75) using etiological and CCRS reference standards. Using pleural tissue samples for TB-LAMP can provide faster results, allowing an early and accurate TB diagnosis in both etiology and clinically diagnosed TB patients.

Against the etiological diagnosis standard, we identified the sensitivity of TB-LAMP was 77.8%, which was significantly better than that of MGIT 960 culture at 10.6%, which is currently regarded as a gold stander diagnostic test in China. The reason for the low sensitivity of MGIT 960 culture could be attributed to the fact that the patients' fibers were thickened due to TB empyema, and the bacterial load of pleural tissue samples was lowered subsequently. In the meantime, MGIT 960 culture takes more than 2–3 weeks, to find a method for the diagnosis of TB empyema patients rapidly is of great clinical importance. As an earlier diagnosis would reduce the suffering of the patients and could improve their prognosis. NAATs are a rapid molecular technique to detect small amounts of MTB genetic materials. While most NAATs require a separate DNA extraction step, TB-LAMP requires only the use of a DNA extraction solution (Loopamp PURE DNA Extraction Kit). In addition, amplification results can be read with the naked eye under UV light. Our findings suggested that TB-LAMP achieved high diagnostic sensitivity in TB empyema using pleural tissue samples. TB-LAMP has great potential in the early diagnosis of TB empyema, which was proven to be efficient, accurate, and simple.

Because of the low sensitivity of MTB culture in TB empyema, we also assessed the diagnostic performance against CCRS. Our results showed that when using CCRS as a reference, the sensitivity of TB-LAMP reached 67.2%, which was significantly higher than that of MGIT 960 culture (*P* < 0.05). The sensitivity of TB-LAMP was 67.2% lower than that of pathology at 77.7% (*P* < 0.05). In our previous study, which also evaluated the positivity rate of pleural biopsy at endoscopy in 113 patients with pleural tuberculosis, 71 patients (71/113 62.8%) were positive for caseous granuloma, a result similar to the findings of the present study (19). TB-LAMP has an additional advantage over pathology in areas with a high burden of TB. Because of the high TB prevalence, granulomatous changes in pleural tissue are usually considered to be TB, but in rare cases, granulomatous changes may also be due to other diseases. There would be no false positive report using TB-LAMP under the same situation. Therefore, a positive TB-LAMP result increases our confidence in the diagnosis of TB than using pathology alone.

Furthermore, TB-LAMP results may be known within a couple of hours after the surgery, as compared to 3 to 5 days through histopathology. This helps the clinicians to start anti-tubercular therapy early, often on the same day. Taken together, our findings, together with previous studies, suggest that TB-LAMP assay may be a promising test for the diagnosis of TB empyema.

Our result is in line with the conclusion made by a systematic review. It was demonstrated that TB-LAMP had moderate sensitivity (pooled sensitivity 77.7%, 95% CI 71.2–83.0%) and high specificity (98.1%, 95% CI 95.7–99.2%) when using the most stringent culture-based reference standard, therefore, has the potential to be a useful diagnostic test for pulmonary TB (20). The improved sensitivity similar specificity of our study was also in line with a previous study using TB-LAMP for extrapulmonary TB (EPTB) diagnosis (sensitivity and specificity of LAMP against culture was 85.71 and 88.89%; against CRS was 80 and 88.6% respectively) (21). In our study, the combination of pathology with TB-LAMP showed that TB-LAMP was a powerful tool for detecting MTB infection in patients with stage II or III TB empyema, presenting a sensitivity of 84.7%, NPV of 67.3%, a specificity of 96.0%, and PPV of 98.5% when CCRS was used as a reference. This is in line with previous research carried out by Sreedeep et al., who concluded that the combination of TB-LAMP and culture would increase the sensitivity significantly according to CCRS (21). In our study, the performance of combining pathology and TB-LAMP was also significantly better than that of any single test for TB empyema. This could also be demonstrated by the respective ROC curves and AUC values (pathology and TB-LAMP in combination: AUC of 0.904; pathology alone: AUC of 0.782; TB-LAMP alone, AUC: 0.710). As these results were similar to combining pathology, MGIT 960 culture, and TB-LAMP, we concluded that combining pathology and TB-LAMP is a more rational diagnostic algorithm for TB empyema in Chinese patients.

False-positive results were reported before, most likely due to the dead MTB cell/cell materials were still present after anti-tuberculosis treatment, as molecular tests cannot differentiate between dead and alive target organisms. Patients with a history of anti-tuberculosis treatment may be wrongly diagnosed as active TB patients (11). To avoid potential confounding effects, we excluded patients who were taking anti-TB treatment. In our study, three patients with suspected previous tuberculous pleural infection diagnosed pleural malignant tumor presented with empyema and had a positive TB-LAMP result. As a result, positive results from NAATs-based assays in patients with previous pleural TB should be carefully evaluated.We also focused on this issue and found no significant difference on the positive rate among these three methods after stratifiying patients according to anti-TB therapy. However, our results might be tempered by limited sample size, therefore, future research is warranted.

Our study has the following limitations.

Our findings apply only to patients with stage II/III TB empyema who underwent pleural decortication, which must be performed in a qualified hospital by experienced surgeons. This study is retrospective, and there is no way to compare the differences between the different parts of the samples. A prospective study with more detailed planning could obtain more accurate information on the yield of TB-LAMP on pleural tissue samples.

## Conclusion

With good sensitivity and specificity, TB-LAMP has great potential in the clinical diagnosis of pleural tissue. Our findings provide insights for optimizing diagnostic algorithms for stage II/III TB empyema diagnosis.

## Data Availability Statement

The original contributions presented in the study are included in the article/Supplementary Material, further inquiries can be directed to the corresponding author.

## Ethics Statement

The studies involving human participants were reviewed and approved by Medical Ethics Committee of Shenyang Chest Hospital (KYXM-2019-010-01). Written informed consent to participate in this study was provided by the participants or their legal guardian/next of kin. Written informed consent was obtained from the individual(s), and the minor(s)' legal guardian/next of kin, for the publication of any potentially identifiable images or data included in this article.

## Author Contributions

YC, HT, and YR had full access to all of the data in the study and take responsibility for the integrity of the data, the accuracy of the data analysis, and critical revision of the manuscript for important intellectual content. YC, CL, LF, and YR: concept and design. YC, JZ, QH, CL, and LF: acquisition, analysis, or interpretation of data. YC and CL: drafting of the manuscript. CL: statistical analysis. All authors contributed to the article and approved the submitted version.

## Conflict of Interest

The authors declare that the research was conducted in the absence of any commercial or financial relationships that could be construed as a potential conflict of interest.

## Publisher's Note

All claims expressed in this article are solely those of the authors and do not necessarily represent those of their affiliated organizations, or those of the publisher, the editors and the reviewers. Any product that may be evaluated in this article, or claim that may be made by its manufacturer, is not guaranteed or endorsed by the publisher.
