# Supplementary material for: Performance of TB-LAMP in the Diagnosis of Tuberculous Empyema Using Samples Obtained From Pleural Decortication
Source: Front Med (Lausanne). 2022 Jun 29;9:879772. doi: 10.3389/fmed.2022.879772 (PMC9278273; doi:10.3389/fmed.2022.879772)
Supplement: Supplementary file 1 [file Data_Sheet_1.docx]

**Diagnostic accuracy of the TB-LAMP, MGIT960 culture and pathology as single tests in different anti-TB treatment history**

| **Diagnostic assay** | **History of anti-TB treatment** | **n/N** | **Estimate (95% CI)** | **P值** |
| --- | --- | --- | --- | --- |
| Pathology | Yes | 42/70 | 60.0(47.6-71.3) | 0.779 |
|  | No | 136/234 | 58.1(51.5-64.5) |  |
| MGIT 960 | Yes | 5/70 | 7.1(2.7-16.6) | 0.725 |
|  | No | 14/234 | 6.0(3.4-10.0) |  |
| TB-LAMP | Yes | 39/70 | 55.7(43.4-67.4) | 0.437 |
|  | No | 118/234 | 50.4(43.9-57.0) |  |

| **Diagnostic assay** | **Gender** | **n/N** | **Estimate (95% CI)** | **P值** |
| --- | --- | --- | --- | --- |
| Pathology | Male | 128/220 | 58.2(51.3-64.7) | 0.832 |
|  | Female | 50/84 | 59.5(48.2-69.9) |  |
| MGIT 960 | Male | 13/220 | 5.9(3.3-10.1) | 0.691 |
|  | Female | 6/84 | 7.1(2.9-15.5) |  |
| TB-LAMP | Male | 116/220 | 52.7(45.9-59.4) | 0.541 |
|  | Female | 41/84 | 48.8(37.8-59.9) |  |

**Diagnostic accuracy of the TB-LAMP, MGIT960 culture and pathology as single tests in different genders**
